# The Impact of Duration of Treatment on Reported Time-to-Onset in Spontaneous Reporting Systems for Pharmacovigilance

**DOI:** 10.1371/journal.pone.0068938

**Published:** 2013-07-15

**Authors:** Ghazaleh Karimi, Kristina Star, G. Niklas Norén, Staffan Hägg

**Affiliations:** 1 Uppsala Monitoring Centre, WHO Collaborating Centre for International Drug Monitoring, Uppsala, Sweden; 2 Department of Public Health and Caring Sciences, Uppsala University, Uppsala, Sweden; 3 Department of Mathematics, Stockholm University, Stockholm, Sweden; 4 Department of Drug Research/Clinical Pharmacology, Linköping University, Linköping, Sweden; Chancellor College, University of Malawi, Malawi

## Abstract

Within pharmacovigilance, knowledge of time-to-onset (time from start of drug administration to onset of reaction) is important in causality assessment of drugs and suspected adverse drug reactions (ADRs) and may indicate pharmacological mechanisms involved. It has been suggested that time-to-onset from individual case reports can be used for detection of safety signals. However, some ADRs only occur during treatment, while those that do occur later are less likely to be reported. The aim of this study was to investigate the impact of treatment duration on the reported time-to-onset. Case reports from the WHO Global ICSR database, VigiBase, up until February 5^th^ 2010 were the basis of this study. To examine the effect of duration of treatment on reported time-to-onset, angioedema and hepatitis were selected to represent short and long latency ADRs, respectively. The reported time-to-onset for each of these ADRs was contrasted for a set of drugs expected to be used short- or long-term, respectively. The study included 2,980 unique reports for angioedema and 1,159 for hepatitis. Median reported time-to-onset for angioedema in short-term treatments ranged 0-1 days (median 0.5), for angioedema in long-term treatments 0-26 days (median 8), for hepatitis in short-term treatments 4-12 days (median 7.5) and for hepatitis in long term treatments 19-73 days (median 28). Short-term treatments presented significantly shorter reported time-to-onset than long-term treatments. Of note is that reported time-to-onset for angioedema for long-term treatments (median value of medians being 8 days) was very similar to that of hepatitis for short-term treatments (median value of medians equal 7.5 days). The expected duration of treatment needs to be considered in the interpretation of reported time-to-onset and should be accounted for in signal detection method development and case evaluation.

## Background

Information on when in time an adverse drug reaction (ADR) is most likely to occur in relation to when a drug therapy is initiated (denoted time-to-onset in this study) is of clinical importance. It helps patients and health care professionals to be aware of when they should be particularly vigilant in following up on new prescriptions. Within pharmacovigilance, time-to-onset is one of the most fundamental criteria when assessing the likelihood of a causal relationship between a suspected ADR and a drug. Time-to-onset in case series analysis is crucial in order to determine whether a typical pattern exists, which can provide clues to the pharmacological mechanisms behind the ADR.

There have been suggestions on using time-to-onset data from large collections of individual case reports to develop methods for detecting safety signals based on statistical analysis [1,2]. Maignen et al. used methods from survival analysis to fit mathematical models to the distribution of reported time-to-onset for different drug-ADR pairs [1]. Van Holle et al. instead identified drug-ADR pairs with empirical distributions of reported time-to-onset that deviate from other ADRs with the drug of interest and of the ADR of interest with other drugs [2]. However, time-to-onset information from reports is known to be heterogeneously collected [3]. Additionally, such large collections include a multitude of drugs with various durations of use, which might affect any comparison of aggregated reported time-to-onset, if not explicitly adjusted for. Some adverse reactions only occur during treatment. In general, adverse events are less likely to be suspected and reported after treatment ends. Thus, the observed time-to-onset may depend on both the latency of the event and the expected duration of treatment. The aim of this study was to investigate the impact of differences in duration of treatment on the reported time-to-onset of angioedema and hepatitis in a spontaneous reporting system, to inform future signal detection and analysis based on aggregated reported time-to-onset.

## Methods

In order to evaluate the relationship between duration of treatment and reported time-to-onset, we contrasted the distribution of reported time-to-onset of angioedema (with an expected short latency) and hepatitis (with an expected longer latency) for a set of drugs expected to be used as short-term treatments with a set of drugs expected to be used as long-term treatments.

### VigiBase

The WHO Global ICSR database, VigiBase [4], holds reports on suspected ADRs from more than 100 countries around the world. The reporting countries are members of the WHO Programme for International Drug Monitoring and contribute reports from their respective national ADR reporting system. This study was based on reports up until February 5th 2010, when a total of 4,978,565 reports were accumulated in VigiBase. The reported drugs are encoded using the WHO Drug Dictionary Enhanced, which uses the WHO Anatomical Therapeutic Chemical (ATC) classification. The selected drugs were studied at the preferred base level. ADRs are encoded in the WHO Adverse Reactions Terminology (WHO-ART) and the Medical Dictionary for Regulatory Activities (MedDRA) in parallel. The latter is built up by five levels of hierarchy [5], and this study was carried out at the level of MedDRA preferred terms (PT), which is the fourth of those.

### Reported time-to-onset

In the context of this study we refer to the time span between reported start of drug therapy and reported onset of the ADR as the reported time-to-onset (RTTO), acknowledging the variable information that this may represent [1,3].

### Selection of adverse drug reactions

To examine the effect of duration of treatment on RTTO, angioedema and hepatitis were selected as two well-studied ADRs, providing examples of mechanistically diverse conditions which differ in expected latency.

Angioedema is typically an abrupt ADR, classified according to the underlying pathophysiology [6]. Allergic histamine-mediated and pseudoallergic cyclooxygenas inhibition-mediated angioedema occur within hours of exposure to the offending drug, the former being dependent on prior sensitization. However, bradykinin-mediated angioedema associated with angiotensin-converting enzyme inhibitors (ACEI) is an exception, as it may occur during the first week of therapy or be delayed up to several months [7]. None of the other drugs studied in our analysis (see section 2.4) have been documented to induce angioedema by a bradykinin-mediated mechanism. Therefore the time-to-onset for angioedema is expected to be short for the majority of the studied drugs.

Drug induced hepatitis is commonly divided into intrinsic and idiosyncratic reactions [8], where the dose dependent reactions in the intrinsic category mainly are caused by acetaminophen toxicity [9,10]. In contrast, idiosyncratic hepatitis has no immediate relationship to dose, although a dose threshold has been suggested [11,12]. Among the proposed underlying mechanisms, immune-mediation is suggested to cause liver injury with latencies as short as 1-8 weeks, while non-immune mediated reactions may take up to one year [9]. The median time-to-onset of drug-induced liver injury was found to be 42 days (range 20-117 days) in a prospective observational study [13]. However, biochemical signs of drug induced hepatitis by acetaminophen is known to present within 24 hours from intoxication [14].

The basis for our analysis were the MedDRA Preferred Terms (PTs) describing the two selected ADRs: ‘Angioedema’ and ’Hepatitis’. These PTs contain a range of Lowest Level Terms (LLTs) reflecting the same medical concept as the corresponding PT, expressed by synonyms and lexical variants [15]. After a review of all LLTs for both of the PTs in MedDRA, LLTs indicating that the ADRs had been aggravated were excluded e.g. ‘Angioedema aggravated’, ‘Angioneurotic edema aggravated’, ‘Angioneurotic oedema aggravated’ and ‘Hepatitis aggravated’. In addition, LLTs indicating too unspecific conditions such as ‘Edema vascular’, ‘Oedema vascular’ and ‘Hepatitis in other infectious diseases classified elsewhere’ were removed from the dataset, as well as LLTs referring to a specific inappropriate subtype of the ADR e.g. ‘C1 esterase deficiency acquired’ and ‘Syncytial giant cell hepatitis’. An overview of the studied MedDRA PTs and included LLTs after applying the exclusion of the above mentioned are presented in Table S1.

### Selection of short-versus long-term treatments

A review of the top reported drugs for angioedema and hepatitis was performed. Among these, six substances (or combined substances reported as combination products) representing expected short- and six representing long or continuous duration of treatment were selected, with a known potential of causing both studied ADRs. Short-term treatments were considered therapies with a recommended duration of therapy of three weeks or less, while long-term treatments were therapies with a recommended duration of therapy of three months or longer. Each included drug was checked for these durations of treatment according to the literature [16]. Only substances reported for both studied ADRs were chosen, to enable a comparison of the impact of duration of treatment on the RTTO. Special care was taken to select substances from different pharmacological classes and thus mechanisms of ADR induction. Drugs chosen as representatives of short-term treatments were erythromycin (macrolid), ibuprofen (nonsteroidal anti-inflammatory drug, NSAID), clavulanate potassium/amoxicillin trihydrate (penicillin/ β-lactamase inhibitor), sulfamethoxazole/ trimethoprim (sulfonamide/synthetic antibiotic agent), paracetamol (acetaminophen, anilid) and ciprofloxacin (fluoroquinolone). The representative substances chosen for long-term treatments were enalapril (angiotensin-converting enzyme inhibitor, ACEI), fluoxetine (selective serotonin reuptake inhibitor, SSRI), isotretinoin (retinoid), methotrexate (antimetabolite), simvastatin (HMG-CoA reductase inhibitor), and ticlopidine (platelet aggregation inhibitor).

### Data extraction and criteria

Reports containing the defined terms were extracted from the database and subject to the following selection criteria. Firstly we focused exclusively on reports with a single suspected drug, to circumvent the complexity of multiple suspected drugs with different start dates. Secondly, suspected duplicates were removed using a previously described probabilistic record matching algorithm [17]. Furthermore, reports with imprecise relevant dates, i.e. lacking specified day or month for start of drug treatment or onset of suspected ADR(s) as well as reports with potentially imprecise dates that referred to 01 and/or 15 of a month were excluded. The latter dates are known to sometimes represent estimated dates by the national pharmacovigilance centre, where only the month or the year is known. Finally, to reduce the impact of data quality issues, reports with unspecified gender or age were excluded in addition to reports with negative RTTO or RTTO exceeding patient age.

### Ethics statement

De-identified individual case reports have been routinely collected as a public health service internationally since 1968, through the WHO Programme for International Drug Monitoring. The protection of the identity of the patient and the reporter has been routine from the outset.

### Statistical analysis

The reported time to onset for different drugs with angioedema and hepatitis was visualized in box plots, using the “bwplot” function in the lattice package of R [18]. In these plots, the box demarks the interquartile range, the medians are marked with a dot, and whiskers extend to the most extreme data point that falls within 1.5 times the interquartile range of either end of the box; outliers beyond the whiskers are not displayed. Comparisons between long- and short-term treatments in the reported time-to-onset for each ADR were made with non-parametric Mann-Whitney tests. The null hypothesis is that there is no difference in reported time-to-onset between the two groups of drugs, for each respective ADR. The “wilcox. test” R function for a two-tailed and unpaired Wilcoxon rank sum test [19] was used, which is equivalent to the Mann-Whitney test [20]. Formal survival analysis was not possible, lacking information on the number of patients at risk at a given point in time after initiation of treatment.

## Results

Under the exclusion criteria specified for the PTs ‘Angioedema’ and ‘Hepatitis’ (see Table S1 for studied LLTs), 36,654 ICSRs with single suspected drugs and removed suspected duplicates were retrieved for this study. The proportion of excluded reports with imprecise (i.e. partly missing) or potentially imprecise (i.e. start or onset day 01 and/or 15) RTTO was 41% for hepatitis and 24% for angioedema. Out of these, the potentially imprecise dates accounted for 25% for hepatitis compared to 16% for angioedema. Other applied exclusion criteria for assuring data quality (specified in section 2.5) only affected 4% of the data on hepatitis versus 6% on angioedema.

**Figure 1:**
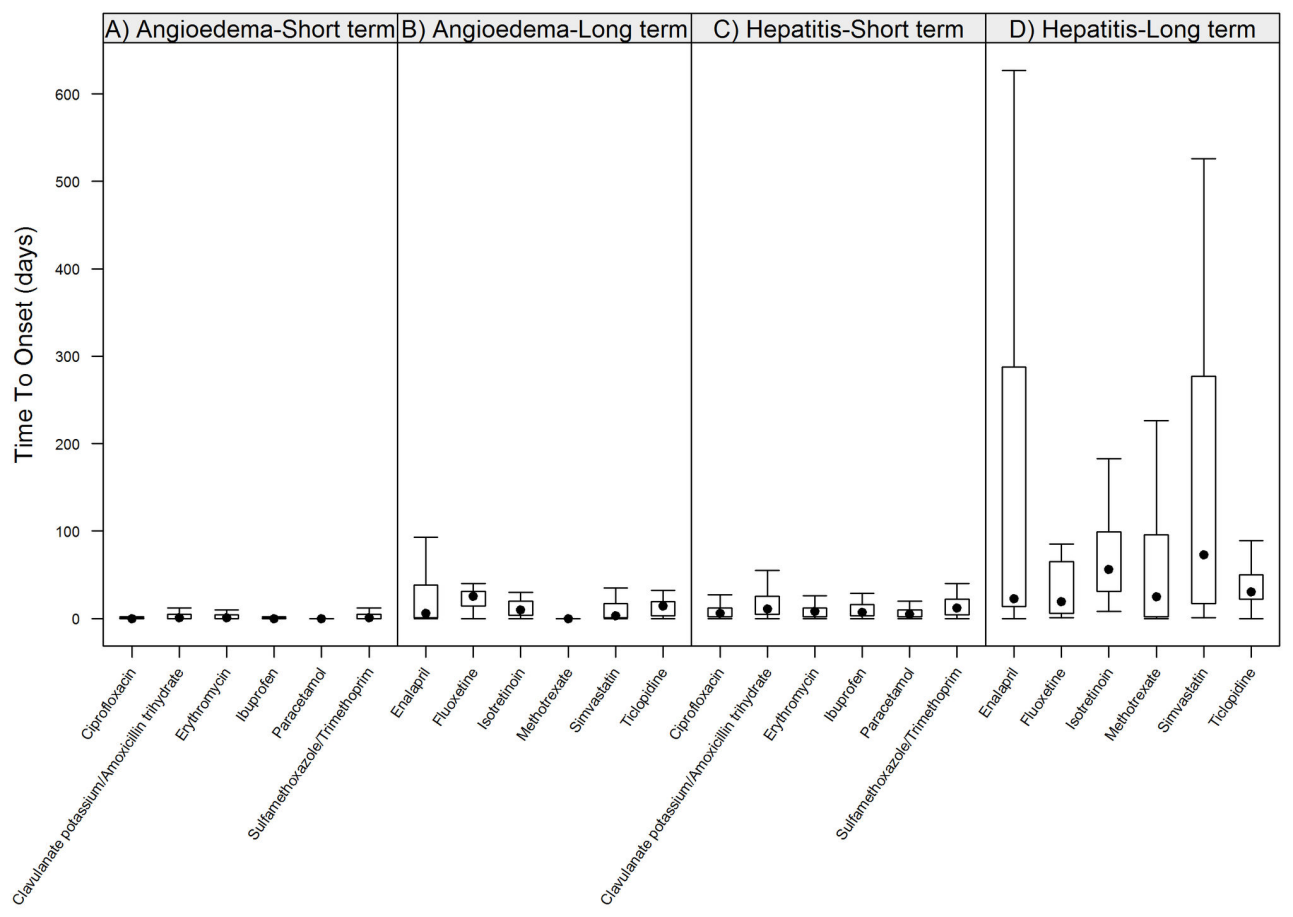
Reported time-to-onset for short-versus long-term treatments for angioedema and hepatitis, respectively. Drugs with expected long-term treatments (panels B and D) generally presented with longer RTTO than drugs with expected short-term treatment (panels A and C). In fact, the patterns of RTTO of angioedema for long-term treatments were very similar to the patterns of RTTO of hepatitis for short-term treatments (panels B and C).

**Figure 2:**
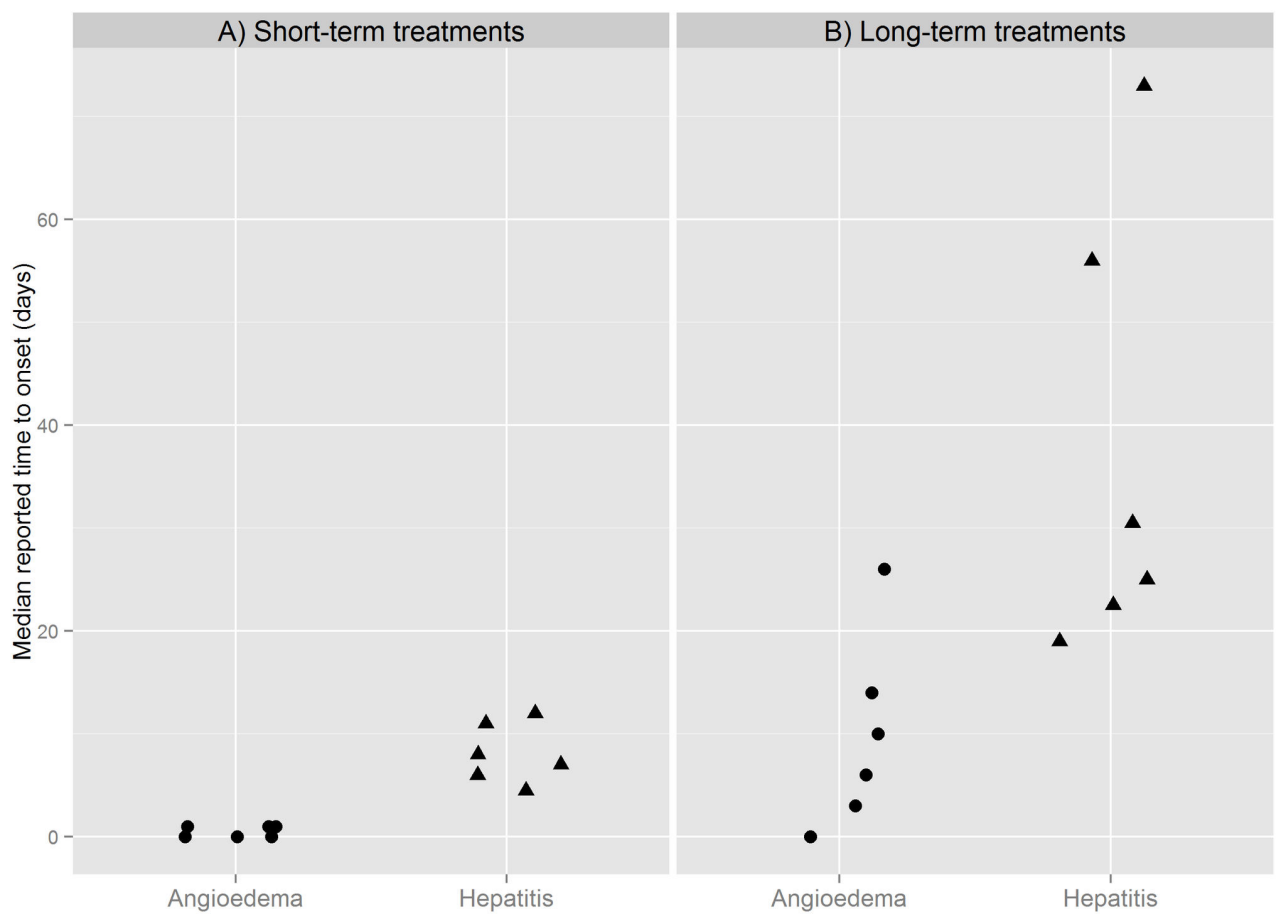
Median RTTO for short-versus long-term treatments for angioedema and hepatitis, respectively. The plot visualises the overlap of RTTO medians of short-term treatments for hepatitis with those of long-term treatments for angioedema. The median value of medians for RTTO of angioedema with long-term treatments was 8 days and the corresponding median for hepatitis with short-term treatments was 7.5 days.

The analysis of RTTO for the selected short- and long-term treatments included 4,139 reports, the demographics of which are presented in Table S2. As groups, drugs with expected long-term treatments presented significantly longer RTTO than drugs with expected short-term treatments for both angioedema and hepatitis, (with significance levels << 0.001 for both ADRs). In fact, the patterns of RTTO of angioedema for long-term treatments were very similar to the patterns of RTTO of hepatitis for short-term treatments, see Figure 1. Median RTTO for angioedema with long-term treatments ranged 0-26 days, with a median value at 8 days. Fluoxetine presented the highest median RTTO for angioedema among the studied drugs with a latency of 26 days. The corresponding range of median RTTO for hepatitis with short-term treatments was 4-11 days, with a median value at 7.5 days, see Figure 2.

In order to investigate the effect of excluding potentially imprecise dates (i.e. start or onset day 01 and/or 15) from the dataset, a sensitivity analysis including the reports with potentially imprecise RTTO for angioedema and hepatitis with the twelve evaluated drugs was conducted. However, it demonstrated that the effect on outcome RTTO was negligible (results not presented here).

## Discussion

Our results show that duration of treatment has a significant impact on the distribution of reported time-to-onset in spontaneous reporting systems in pharmacovigilance. Notably, the median of the median RTTO of ‘angioedema’ for long-term treatments (8 days) exceeded that of ‘hepatitis’ for short-term treatments (7.5 days). In addition, the RTTO for hepatitis for the short-term treatments (range 4-11 days) is substantially shorter than that observed for an overlapping set of drugs in the prospective study by Chalasani et al. (range 20-117 days). These findings suggest that aggregated time-to-onset for short-term treatments cannot be reliably evaluated in spontaneous reporting systems without taking into account the expected duration of treatment. The shorter than expected RTTO for hepatitis with short-term treatments must be interpreted bearing in mind that adverse events are less likely to be reported as suspected ADRs after the end of treatment. Thus, this does not rule out the possibility that they represent causal associations. Whereas we anticipated short-term treatments to exhibit shorter than expected reported time-to-onset, we had not anticipated the longer than expected reported time-to-onset of angioedema for the long-term treatments. Fluoxetine for example presented a median RTTO of 26 days, with RTTO exceeding 30 days for more than 25% of the reports. While long time-to-onsets may be associated with imprecise information more often, our sensitivity analysis indicated that if such an effect was present it did not have a major impact on our results.

When categorizing drugs into short term (up to 3 weeks) and long term (3 months or more) treatment groups, some drugs were not easily classified. The non-steroidal anti-inflammatory drug ibuprofen for instance was classified as a short term analgesic, while it is known to be used long term in the treatment of chronic inflammations for example rheumatoid arthritis and arthrosis [16]. In an attempt to circumvent the possibility of varying durations of treatments within each drug, a restriction was made to reports with a specified indication for the suspected drug. However, this approach proved intractable, as the numbers of reports on either angioedema or hepatitis for each drug were too low to allow for robust analysis. Despite the special care taken in selecting drugs from different pharmacological classes, the panel of drugs for short term treatments was dominated by antibacterials (ATC J01). The broader group of antimicrobials (J) has previously been observed as the single largest class of agents causing drug induced liver injury [13]. Nevertheless, two of six short term treatments did not belong to the ATC-class (J), confirming that the short observed RTTO were not likely due to a class effect.

Confounding by underlying infection (viral hepatitis) cannot be excluded as an explanation for the shorter RTTO for short term treatments. The studied treatments consist of antibacterial drugs, with the exception of ibuprofen (non-steroidal anti-inflammatory drug) and paracetamol (anilid). However, the RTTO for the two non-antibacterials are also short and it is unlikely that the majority of reports for the antibacterials should be for patients with a viral infection misdiagnosed as bacterial.

VigiBase pools data originating from a range of professional settings, geographical regions and time periods with varying legislation and tradition of reporting. The diversity of reporters includes health care professionals, patients and pharmaceutical companies. Different criteria and routines for receiving, recording and managing reports prior to submission to VigiBase [4] result in an intrinsically heterogeneous collection of data where it is likely that the RTTO varies with the type of reporter. A suggestive time-to-onset is a major argument for suspecting and reporting an adverse event as a suspected ADR. However, the level of suspicion for the reported drug-ADR pairs in VigiBase is not specified for all reports and will vary within the dataset.

A previously discussed phenomenon that may affect the output of aggregated time-to-onset information is how the reporter has defined the onset date of the ADR, which can be recorded as when the first symptom started, when the diagnosis was made, or when the ADR became serious and thereby reportable by the pharmaceutical companies [3]. As ADRs may develop certain seriousness criterion over time, e.g. hospitalization, the RTTO will inherently be longer than the actual occurrence of the ADR. The generally quick onset of angioedema requiring hospitalization would be less likely influenced by this reporting bias, whilst for hepatitis the latency would be more likely prolonged by this bias rather than shortened, as was the tendency for both short-term and long-term treatments in this study.

Our study of the influence of treatment duration on RTTO was limited to two ADRs: angioedema and hepatitis. Further work is required to evaluate to what extent the phenomenon affects other ADRs. Treatments without duration, i.e. vaccinations or anesthesia, obviously follow a different pattern where the likelihood that an adverse event is reported as a suspected ADR can be expected to decrease more gradually from the time of treatment.

## Conclusion

The expected duration of treatment needs to be considered in the interpretation of reported time-to-onset and should be accounted for in signal detection method development and case evaluation.

## Supporting Information

Table S1 MedDRA terms. List of Lower Level Terms (LLTs) for the two studied MedDRA Preferred Terms (PTs) Angioedema and Hepatitis included in the analysis.
(DOCX)

Table S2 Report demographics. Number of reports in the WHO global ICSR database VigiBase included for each studied drug-ADR pair.
(DOCX)
