# Supplementary material for: The Impact of Duration of Treatment on Reported Time-to-Onset in Spontaneous Reporting Systems for Pharmacovigilance
Source: PLoS One. 2013 Jul 15;8(7):e68938. doi: 10.1371/journal.pone.0068938 (PMC3711907; doi:10.1371/journal.pone.0068938)
Supplement: Table S1 — List of Lower Level Terms (LLTs) for the two studied MedDRA Preferred Terms (PTs) Angioedema and Hepatitis included in the analysis. (DOCX) [file pone.0068938.s001.docx]

Table S1. **MedDRA terms**

| MedDRA Preferred Terms | MedDRA Lower Level Terms^[[1]](#footnote-1)^ |
| --- | --- |
| Angioedema |  |
|  | Acute angio oedema |
|  | Angio-edema |
|  | *Angio-oedema* |
|  | Angioedema |
|  | Angioneurotic edema |
|  | *Angioneurotic edema, not elsewhere classified* |
|  | Angioneurotic oedema |
|  | Edema angioneurotic |
|  | Edema Quincke's |
|  | Giant hives |
|  | Giant urticaria |
|  | *Hives giant* |
|  | Quincke's edema |
|  | Quincke's oedema |
|  | Urticaria giant |
|  | Allergic angioedema |
|  | Acute angio edema |
|  | Oedema angioneurotic |
|  | Oedema Quincke's |
| Hepatitis |  |
|  | Drug-induced hepatitis |
|  | Hepatitis |
|  | Hepatitis allergic drug-induced |
|  | Hepatitis drug-induced |
|  | *Hepatitis nonicteric* |
|  | Hepatitis non-specific |
|  | Hepatitis NOS |
|  | *Hepatitis reactive non-specific* |
|  | Hepatitis symptom |
|  | Hepatitis, unspecified |
|  | *Nonspecific hepatitis* |
|  | *Hepatitis non-icteric* |

List of Lower Level Terms (LLTs) for the two studied MedDRA Preferred Terms (PTs) Angioedema and Hepatitis included in the analyses.

1. Lower level terms in italics: No reports found for the LLTs matching the inclusion criteria. [↑](#footnote-ref-1)
