# Supplementary material for: The Impact of Duration of Treatment on Reported Time-to-Onset in Spontaneous Reporting Systems for Pharmacovigilance
Source: PLoS One. 2013 Jul 15;8(7):e68938. doi: 10.1371/journal.pone.0068938 (PMC3711907; doi:10.1371/journal.pone.0068938)
Supplement: Table S2 — Number of reports in the WHO global ICSR database VigiBase included for each studied drug-ADR pair. (DOCX) [file pone.0068938.s002.docx]

Table S2. **Report demographics**

| Substance(s) | Angioedema | Hepatitis |
| --- | --- | --- |
| Ciprofloxacin | 135 | 60 |
| Amoxicillin/clavulanate | 153 | 215 |
| Erythromycin | 81 | 207 |
| Ibuprofen | 694 | 36 |
| Paracetamol | 188 | 75 |
| Sulfamethoxazole/trimethoprim | 482 | 186 |
| Enalapril | 816 | 24 |
| Fluoxetine | 88 | 24 |
| Isotretinoin | 19 | 41 |
| Methotrexate | 5 | 38 |
| Simvastatin | 49 | 54 |
| Ticlopidine | 33 | 84 |
| Sum | 2743 | 1044 |

Number of reports in the WHO global ICSR database VigiBase included for each studied drug-ADR pair.
